# Spatial decision dynamics during wayfinding: intersections prompt the decision-making process

**DOI:** 10.1186/s41235-018-0098-3

**Published:** 2018-05-02

**Authors:** Tad T. Brunyé, Aaron L. Gardony, Amanda Holmes, Holly A. Taylor

**Affiliations:** 1 Center for Applied Brain & Cognitive Sciences, Tufts University, 200 Boston Ave., Suite 3000, Medford, MA 02155 USA; 2 U.S. Army Natick Soldier Research, Development, and Engineering Center, RDNS-SEW-THC, 15 General Greene Ave, Natick, MA USA; 3 Department of Psychology, Tufts University, 490 Boston Ave., Medford, MA USA

**Keywords:** Navigation, Decision-making, Wayfinding, Spatial cognition

## Abstract

Intersections are critical decision points for wayfinders, but it is unknown how decision dynamics unfold during pedestrian wayfinding. Some research implies that pedestrians leverage available visual cues to actively compare options while in an intersection, whereas other research suggests that people strive to make decisions long before overt responses are required. Two experiments examined these possibilities while participants navigated virtual desktop environments, assessing information-seeking behavior (Experiment 1) and movement dynamics (Experiment 2) while approaching intersections. In Experiment 1, we found that participants requested navigation guidance while in path segments approaching an intersection and the guidance facilitated choice behavior. In Experiment 2, we found that participants tended to orient themselves toward an upcoming turn direction before entering an intersection, particularly as they became more familiar with the environment. Some of these patterns were modulated by individual differences in spatial ability, sense of direction, spatial strategies, and gender. Together, we provide novel evidence that deciding whether to continue straight or turn involves a dynamic, distributed decision-making process that is prompted by upcoming intersections and modulated by individual differences and environmental experience. We discuss implications of these results for spatial decision-making theory and the development of innovative adaptive, beacon-based navigation guidance systems.

## Significance

We study the dynamics of pedestrian decision-making during urban wayfinding, with implications for the development of adaptive navigation guidance systems. Results demonstrate that pedestrians begin making turn decisions far before reaching an intersection and this pattern is dictated by spatial skills and experience with the environment, informing when, where, and for whom future navigation systems might cue behavior.

### Background

Wayfinding involves deliberate navigation between two or more points of interest and can take place in both familiar (e.g. near home) and unfamiliar (e.g. on vacation) environments. Though ubiquitous in daily life, wayfinding is exceedingly complex and involves a dynamic interplay between the perceived environment and multiple cognitive processes. Indeed, wayfinders form associations between elements of the environment, think critically regarding where to go and what to do, actively engage in spatial decision-making along the way, and ultimately select movement sequences to guide them toward a destination (Wiener, Büchner, & Hölscher, 2009). How spatial decisions are made and which aspects of the environment are crucial for supporting them are topics receiving considerable attention. This is due to both the theoretical implications for models of wayfinding and spatial decision-making and for establishing principles for the portrayal of next-generation navigation guidance (Cosgun, Sisbot, & Christensen, 2014; Hwang & Ryu, 2010; Van Erp, Van Veen, Jansen, & Dobbins, 2005). For instance, beacon-based navigation aids can provide pedestrians information regarding the general direction and distance to a waypoint, efficiently guiding them to a destination without the attention and memory disadvantages of turn-by-turn guidance (Brunyé, Moran, Houck, Taylor, & Mahoney, 2016a; Gardony, Brunyé, & Taylor, 2015). A beacon is a landmark or device that marks a fixed location (e.g. a lighthouse or radio beacon) to directly guide a navigator toward a destination (or near a destination (Waller & Lippa, 2007)), and/or provide them with information about their bearing (i.e. the direction to a given object or location; Priyantha, Chakraborty, & Balakrishnan, 2000). Future instantiations of beacon-based guidance systems, for instance on a heads-up display or vibrotactile belt, may adaptively provide wayfinders with spatial information as a function of circumstances; for instance, while the wayfinder is in an intersection, or when displaying signs of disorientation or confusion (Brunyé, Haga, Houck, & Taylor, 2018).

To inform the timing and spatial distribution of adaptive navigation guidance, we must first understand how and when pedestrians make spatial decisions during wayfinding. One recurring theme of extant wayfinding research is the apparent importance of landmarks and intersections for understanding and reasoning about an environment (Janzen, 2006; Lynch, 1960; Presson & Montello, 1988). Landmarks are critically important reference points for orienting and understanding relative location (but see Montello, 2017) and intersections are implicated as critical locations where decisions are made regarding whether to continue forward or turn (Golledge, 1999; Klippel, 2003). If spatial decisions are indeed made within an intersection, then it may be suitable to dynamically cue navigation guidance when a pedestrian enters an intersection; however, if spatial decision-making is relatively protracted and precedes the entrance to an intersection, it may prove advantageous to cue guidance in anticipation of upcoming intersections. To better understand the spatial decision-making process and provide a foundation for answering these applied questions, the present experiments examine choice behavior while participants wayfind between destinations in a virtual city.

### Wayfinding: planning and execution

Several cognitive models of wayfinding have been proposed. The majority of these includes reference to route planning and plan execution and describes an iterative sequence of perceptual and cognitive processes, including decision-making, that unfolds during wayfinding (Spiers & Maguire, 2008). Route planning describes the process of reviewing internal (memory) and/or external (maps) information to plan a sequence of navigation actions from an origin to a destination. During the planning process, several strategies and heuristics have been identified. For instance, planners tend to select paths that: contain fewer decision points (*least-decision-load* strategy (Wiener, Schnee, & Mallot, 2004)); deviate minimally from the overall direction of a goal (*least-angle* strategy (Dalton, 2003)); have a long and straight segment leaving an origin (*initial segment* strategy (Bailenson, Shum, & Uttal, 2000); and head generally south rather than north (*southern route preference* (Brunyé, Mahoney, Gardony, & Taylor, 2010)). Overall, planners appear to identify potential routes that satisfy their goals and then use several (some implicit) strategies to quickly reduce options and settle on a route. Sometimes only a very coarse planning process may occur, for instance, when traveling between relatively distant places and regions (Wiener & Mallot, 2003). Route planning is not always possible to perform before wayfinding, such as in conditions of limited knowledge or information available about the environment. In still other cases, wayfinders will rely upon automated route guidance, passively following a prescribed sequence of turns and distances.

After planning a route, wayfinding itself progresses through the translation of a route plan to physical actions (e.g. walking, driving, or biking in a goal-oriented manner) (Timpf, Volta, Pollock, & Egenhofer, 1992). Passini describes this process as occurring among three phases: (1) retrieving and manipulating spatial information from current and past experiences (including identifying the location of, and direction to, a destination); (2) developing plans for executing the task; and (3) finally executing the plans by transforming them into overt behavior (Passini, 1981). The first two phases can occur either offline before wayfinding, or online in an iterative manner during active wayfinding; the latter is the focus of the present research. A phased emergence of iterative planning, decision-making, and action execution is foundational to many other wayfinding models (Garling, Book, & Lindberg, 1984). Spiers and Maguire (2008) also describe an iterative re-planning process that takes place during wayfinding: seeking expected landmarks and views to cue particular actions (e.g. turn right at the bank) and then re-planning to resolve any expectation violations as the environment is perceived during navigation. During this process, wayfinders may hold a mental image regarding the shape, size, and visual details of particular landmarks (Passini, 1981). They then verify expectations by matching direct perception to the mental image. If expectations are violated, a re-planning processes is engaged (Aginsky, Harris, Rensink, & Beusmans, 1997). In most wayfinding research, there is a strong emphasis on two aspects of the environment: landmarks and intersections. Landmarks are the environmental features that prompt familiarity, resolve locational ambiguity, and cue sequences of actions. Often, wayfinders focus on landmarks positioned within particular intersections, using them both for recognition and to cue appropriate actions (Brunyé et al., 2015; Golledge, 1999; Janzen, 2006; Klippel, 2003). For this reason, intersections are often deemed “decision points” given that they elicit choice behavior during wayfinding.

### Intersections are critical for decisions

During both planning and wayfinding, intersections have been repeatedly implicated as critical decision points, as they force wayfinders to make decisions regarding how to continue their journey. For instance, continue straight, turn around, or turn right or left. Kim and Hirtle (1995) suggested that knowledge of routes is represented as a sequence of intersection-based choice points where procedural decisions must be made, and Lupien et al. elaborate to point out that route complexity is contingent upon the number of intersection-based decision points (Lupien et al., 1998). Furthermore, Janzen notes that intersections are distinct “decision points” or environmental locations where alternative routes can be chosen (Janzen, 2006). There are three primary points to derive from extant research. First, researchers from varied domains, asking a range of research questions, consistently implicate intersections as critical decision points. Second, there is direct reference to decisions being made *at* or *within* the intersection. For instance, in Passini’s discussion of spatial decision-making, it is proposed that only when a wayfinder recognizes a critical landmark or feature while within an intersection will they become aware of which direction to continue traveling (Passini, 1984). And Golledge specifically notes that “decisions are made at each intersection” (Golledge, 1999, p. 103). Finally, some suggest that path segments between intersections are distinctly *non-decision*-related (Klippel, Tappe, & Habel, 2003), suggesting that the decision process does not arise during wayfinding outside of intersections.

Thus, spatial decision-making during wayfinding has been largely conceptualized as a discrete process that occurs when a wayfinder arrives at an intersection. There are some compelling reasons to believe this might be the case. Given that intersection-based behavior is guided by the recognition of critical landmarks or features, entering an intersection is likely to provide the best vantage point for perceiving not only the landmarks within the intersection itself, but also the distant features of each route. For instance, Ruddle et al. found evidence that wayfinders would “look around” while in intersections, gathering information before deciding whether to turn (Ruddle, Payne, & Jones, 1999). We believe this is compelling evidence that turn decisions are made while pedestrians are within an intersection. Arriving in an intersection uniquely affords direct perception of distant landmarks associated with each turn option and can help pedestrians get their bearings by looking at tall buildings or other distal cues. The ability to perceive and track the location of distant cues is important for successful navigation of dense environments where occlusion can temporarily hinder such lines of sight (Arthur & Passini, 1992; Liu et al., 2009). These processes are likely important especially for wayfinding in relatively unfamiliar environments, such as wayfinding from a hotel to a conference center, or later trying to find a restaurant you might have seen along your way. However, as environmental knowledge progresses, a procedural sequence of turns and ultimately an overall environmental representation are learned, suggesting that increased experience will be correlated with decreased reliance on the distant cues perceptible while within intersections. We test this possibility in our second experiment. Also supporting the possibility that turn decisions are only made once a pedestrian arrives within an intersection, domain-general decision-making theory suggests that in some cases people defer a decision until it becomes necessary, particularly in situations involving several alternatives (Shafir, Simonson, & Tversky, 1993). In wayfinding, this might translate to deferring a spatial decision (i.e. continue straight or turn) until positioned in an intersection and a decision becomes necessary.

However, there is also reason to believe that choice behavior observed in intersections might result from a protracted decision-making process that occurs before entering an intersection. Theoretical and empirical support for this possibility comes from some domain-general decision-making research, as well as some wayfinding research. First, some research and theory proposes that people attempt to plan a decision before executing a behavior (Ajzen, 1985; Klein, 1993; Simon, 1959). In other words, most behavior is goal-directed and people decide on an overt behavior (e.g. which way to go) far before when the behavioral response is required. This process may serve to maximize efficiency when the circumstances evolve to require the response (Ouellette & Wood, 1998). Furthermore, a growing body of evidence suggests that overt behavior can index a dynamic evidence accumulation process and observed choice behavior is a result of this process (Koop & Johnson, 2011; Spivey & Dale, 2006). The proposal that visual information is accumulated during decision-making converges with the *reasoned action* portion of the theory of planned behavior (Ajzen, 2011), which emphasizes the importance of online information processing to dynamically and reliably inform decision-making about ongoing and planned behavior. It also converges with tenets of the *recognition-primed decision-making* theory (Klein, 1993), which suggest that decision-makers in real-world contexts continually prepare to initiate action by attempting to commit to an option in advance.

Second, there is some evidence from wayfinding that suggests a protracted decision-making process before entering an intersection and exhibiting choice behavior; critically, however, no research to date has directly examined this possibility. Wayfinding is considered a visually demanding process, with a continuous visual search for distal and proximal landmarks that can unambiguously cue an action (Garling et al., 1984; Passini, 1981, 1984). Spiers and Maguire term this process “visual inspection of environmental features” and suggest that it is one of the most frequent and longest duration processes that occur during wayfinding (Spiers & Maguire, 2008). Assuming a clear line of sight to upcoming intersections, this visual inspection process may be in the service of identifying critical upcoming environmental features that can inform subsequent action (i.e. go straight, turn right, turn left). Stephan Winter proposed the importance of visibility of landmarks in advance of an intersection (Winter, 2003), with landmarks being preferred when they are not only salient but visible before arriving at an intersection. Though he never proposed that a decision itself is made in advance, presumably advance visibility could afford advance decisions. An additional reason to believe that wayfinders may make a decision before arriving at an intersection is provided by Meilinger et al., who assessed performance on a secondary task while participants navigated a desktop virtual environment (Meilinger, Knauff, & Bülthoff, 2008). They found evidence that secondary task accuracy decreased when participants approached an intersection. The authors cautiously suggested that decisions may be made before entering an intersection, but proposed that additional research was needed to support this idea. One goal of the present experiments was to fill this knowledge gap.

### Innovative navigation systems

Theory and empirical research are equivocal and underspecified regarding the nature of spatial decision-making during wayfinding. Specifically, extant research does not effectively describe the extent to which decisions are made within or outside of intersections and what might modulate such a pattern. In addition to this more theoretical question, we also are interested in better understanding the dynamics of spatial decision-making to inform the development of innovative navigation systems. Specifically, this research will provide new insights into when and where in a journey to provide spatial cues to wayfinding pedestrians.

Traditional turn-by-turn navigation systems as commonly found in smartphone apps provide an efficient means for guiding navigation through complex environments. However, they are also highly vulnerable to signal degradation due to urban canyons (i.e. tall buildings blocking satellite signals (Cui & Ge, 2003)), are readily jammed by portable consumer technologies (i.e. GPS denial (Coffed, 2014)), can distract attention away from other tasks (Lee et al., 2014), and severely limit memory for newly experienced environments (Bakdash, Linkenauger, & Proffitt, 2008; Chrastil & Warren, 2013; Gardony et al., 2015; Gardony, Brunyé, Mahoney, & Taylor, 2013). These limitations have encouraged research and development toward identifying novel methods for guiding pedestrian navigation through complex environments. For instance, beacon-based visual navigation aids can show the general direction and distance to a destination, without providing explicit instructions on which routes to take (Brunyé et al., 2016a). In a series of virtual environment navigation experiments, we previously demonstrated the utility of a floating beacon overlaid onto a virtual scene, showing the direction and distance to a goal destination (Brunyé et al., 2016a). In that work, the accuracy of beacon registration predicted the extent to which the beacon increased path efficiency and trust in the system. Similarly, vibrotactile beacons can accomplish a similar function by providing orientation-specific pulses around the torso or leg, guiding a pedestrian without occupying their visual modality (Boll, Asif, & Heuten, 2011; Cosgun et al., 2014; Van Erp et al., 2005). These innovative approaches to navigation guidance are intended to help pedestrians maintain attention on the environment and active engagement in the spatial decision process and thus reduce the negative impact of turn-by-turn navigation on spatial memory formation. They can also rely on inertial navigation data and compass direction to support beacon use during limited satellite reception (Woodman, 2007), for instance in urban canyons, unmapped subterranean areas, contested environments, and disaster-relief zones.

Because navigation systems, like any automated decision aid that offloads cognitive work, can induce complacency and even skill loss with repeated use (Parasuraman, Molloy, & Singh, 1993; Risko & Gilbert, 2016), future systems are likely to provide adaptive cueing based on current circumstances (Bindewald, Miller, & Peterson, 2014). Circumstances may include states of a pedestrian such as cognitive workload (Kaber, Wright, Prinzel, & Clamann, 2005), states of the environment such as complexity, or interactions between pedestrians and environments, such as location and proximity to an intersection (Schmidt, Beigl, & Gellersen, 1999). Regarding proximity to an intersection, pedestrian navigation devices can use an algorithm that provides a maneuver instruction at a variable “warning distance” from an intersection (Tamai & Pai, 1999). In other words, the systems intentionally provide information to enable planning and decision-making regarding an upcoming maneuver (Brown & Laurier, 2012), and when they fail to provide information in a timely manner, navigation is interrupted and a failed maneuver can occur. Next-generation navigation systems using beacon-based guidance may benefit from adaptively cueing pedestrians as a function of external circumstances, such as position relative to an intersection. This would allow the pedestrian to maintain attention on the environment and their tasks (e.g. walking, talking, or work-related tasks), only cueing when and where appropriate. The present research will provide foundational data for informing such a capability.

### The present study

Two experiments examined outstanding questions regarding the nature of spatial decision-making during wayfinding, using a desktop virtual environment. In Experiment 1, we examined information-seeking behavior. Participants were tasked to navigate by virtually walking between a sequence of successive landmarks in an unfamiliar, large-scale virtual city. During each origin-to-destination trial, they could request an on-screen navigation aid (beacon) by pressing the spacebar, showing them the general direction to their destination. The aid could be requested up to five times per trial, but only stayed on the screen temporarily. We examine the spatial distribution of beacon requests, asking whether they are requested within versus outside of intersections and whether they would predict an approach and relatively efficient movement through an intersection. In Experiment 2, we examined the dynamics of heading changes on approach to an intersection. Participants were tasked to navigate the same virtual city, but without beacons available. In other words, they could only rely on environmental learning alone to effectively navigate the city. As wayfinders develop knowledge of the environment over the course of trials, we ask whether movement dynamics on approach to an intersection show evidence of advanced decision-making. Specifically, we ask whether participants dynamically orient themselves in the direction of an upcoming turn before entering an intersection, particularly as they become more familiar with the environment. If wayfinders orient themselves to facilitate an upcoming maneuver, this would provide strong support for the notion that spatial decisions regarding whether to continue straight or turn are made before entering an intersection.

In this study we also conduct some exploratory analyses assessing whether individual differences in spatial abilities and strategies might predict wayfinding behavior. While many typologies of spatial skills exist, one popular way of parsing spatial skills is considering at least spatial visualization, mental rotation, and perspective-taking and transformation abilities (Hegarty & Waller, 2005; Linn & Petersen, 1985; Uttal et al., 2013). Spatial visualization involves sequential manipulations of spatial information, such as seen with a Rubik’s cube or paper-folding task (Kozhevnikov, Motes, & Hegarty, 2007). Mental rotation involves mental spatial transformations of objects around axes of rotation, such as seen in the classic mental rotation task wherein participants compare two three-dimensional objects and imagine rotating one object to match or mismatch a reference object (Shepard & Metzler, 1971). Perspective transformation involves adopting and solving problems from a spatial perspective different from your own; for instance, looking at a map and making turn decisions from an egocentric perspective (i.e. turn left, turn right) (Schultz, 1991). Each of these skills has been associated with navigation behavior; in general, higher spatial skills across these measures predicts higher performance during virtual navigation tasks (Moffat, Hampson, & Hatzipantelis, 1998). Spatial skills are sometimes contrasted with sense of direction and spatial strategies. Sense of direction is an individual’s self-reported assessment of their ability to orient themselves and navigate through large-scale environments (Hegarty, Richardson, Montello, Lovelace, & Subbiah, 2002). Spatial strategies describe the tendency for different people to prefer and rely upon different types of information during navigation (Münzer & Hölscher, 2011; Pazzaglia & De Beni, 2001). For instance, some individuals prefer to think about environments in terms of routes and landmarks along the way, and rely upon those cues while navigating, whereas others prefer to think about map-like (i.e. survey) information (Passini, 1984; Shelton & Gabrieli, 2004). In the present experiments, we ask whether spatial skills, sense of direction, and spatial strategies relate to the use and reliance on spatial guidance during wayfinding, and the tendency to make decisions outside of intersections.

## Experiment 1

In our first experiment, pedestrian navigation was simulated in a large-scale urban desktop virtual environment, with participants navigating point-to-point in the environment (e.g. first to the pet store, then to the laundromat, etc.). To ensure an uninformed search occurred, participants were completely unfamiliar with the environment, including its landmarks, road network, and structure. While wayfinding to each destination, participants could request a floating visual beacon overlaid onto the virtual environment, up to five times during each origin-to-destination trial, to help guide them in the correct direction. If wayfinders seek information to inform a decision before entering an intersection, navigation aid requests should occur in path segments, not in intersections; furthermore, requests should predict an approach to (rather than departure from) an intersection. Finally, if requesting a beacon facilitates a decision before entering an intersection, then executing a behavior (i.e. continuing straight or turning) in the intersection should be faster when a prior beacon was requested versus when it was not. These patterns of wayfinding behavior may be correlated with spatial abilities and strategies, for instance a higher spatial sense of direction might be related to higher path efficiency and perhaps lower beacon reliance overall.

## Experiment 1 method

### Participants and design

Sixty-nine individuals participated for monetary compensation; 19 participants failed to complete the task due to mild simulator sickness (n = 11) or difficulty performing the wayfinding task (i.e. not completing the task within 1.5 h; n = 8). The resulting sample consisted of 38 men and 12 women (*M*_age_ = 23.3). Each participant completed a sequence of 40 wayfinding trials, in a within-participants design. They were provided with several opportunities to view a floating on-screen beacon for 5 s, to show them the general direction of each destination. All procedures were approved by the institutional review boards at Tufts University and the U.S. Army (#1310028).

### Materials

#### Virtual environment

**Fig. 1:**
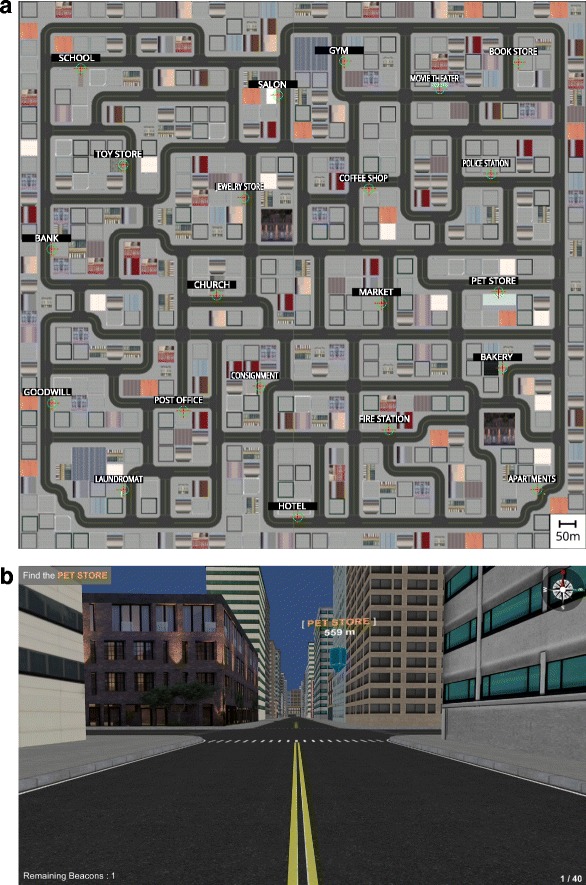
**a**, **b** *Upper panel* depicts an overhead view (north up) of the virtual environment, with labeled landmarks. *Lower panel* depicts a view from within the virtual environment (approaching an intersection), demonstrating the current destination (*Find the Pet Store*), remaining number of beacons (1), trial number (1/40), compass rose, and the floating beacon (PET STORE 559 m)

Using the Unity 3D gaming engine (Unity Technologies; San Francisco, CA, USA), we developed a large-scale urban desktop virtual environment (VE). The environment was approximately square and covered an area of 1.29 km^2^, with 95 intersections and over 500 buildings (Fig. 1a). Twenty-one of the buildings were labeled as target landmarks (e.g. *Bank*) and visually depicted the stated function (e.g. looked like a bank). These 21 landmarks served as goal destinations for the wayfinding task. The Unity 3D gaming engine continuously tracked and outputted participant location in Cartesian space (*x*,*y*), orientation (1–360° yaw), and whether the participant was within or outside of an intersection, over time at approximately 50 Hz. A navigation aid consisted of a floating beacon that indicated the general direction to the destination and its current distance (Fig. 1b). By default, this aid did not appear on the screen unless requested by the participant. When requested, it remained on-screen for 5 s, indicating the general direction of the current trial’s destination. To determine how many times a beacon could be requested within a single trial, we conducted a pilot study (described next).

#### Individual difference measures

We collected data on a range of questionnaires and tasks assessing spatial strategies, ability, and sense of direction. A card rotation task involved a series of 80 trials assessing rotation of two-dimensional figures (Ekstrom, French, Harman, & Dermen, 1976; Hegarty & Waller, 2004). A novel road map task adapted from previous research (Money, Alexander, & Walker, 1965) involved making egocentric turn judgments regarding a path plotted on a map (i.e. turn right/left). The Santa Barbara Sense of Direction questionnaire assessed self-reported spatial sense of direction, which gauges performance on egocentric orienting tasks in large-scale space (Hegarty et al., 2002). We also administered a spatial strategy questionnaire to assess differential reliance on egocentric, survey, and cardinal direction: the Measure of Environmental Spatial Strategies (Münzer & Hölscher, 2011). Finally, we assessed self-reported video game experience by asking participants whether they consider themselves a video gamer, a question adopted from earlier research (Boot, Kramer, Simons, Fabiani, & Gratton, 2008).

### Procedure

#### Pilot experiment

To provide a basis for selecting the appropriate number and duration of beacons available in each trial, a pilot experiment was conducted. Six (*M*_age_ = 22.1 years; 4 men, 2 women) participants attempted to locate a sequence of 40 successive landmarks in the VE. For the first two participants, we allowed two beacons for 3 s duration each; both participants failed to complete the task. For the second two participants, we allowed four beacons for 3 s each; one of the two participants completed the task. Finally, for the final two participants, we allowed five beacons for 5 s duration each and both participants were able to complete the task. Notably, one participant found it much more challenging than the other. Given these results, we determined that the main experiment would provide up to five beacons for 5 s duration each, per trial.

#### Main experiment

Participants visited the laboratory for an approximately 1-h session. Following consent, they performed a brief navigation practice to increase familiarity with the navigation controls. Participants were seated at a 24″ desktop computer monitor (1920 × 1080 resolution), used the *W* key on a standard QWERTY keyboard to walk forward (translational movement), and used the computer mouse to make orientation changes. Walking speed was fixed when the *W* key was pressed, though participants could release the *W* key to pause movement at any time, perhaps to change orientation or look around. The side-to-side movement (*A*, *D*) and backward (*S*) movement keys were disabled. The navigated environment was displayed at full screen and the camera view showed a 90° horizontal visual angle. For practice, they were asked to virtually walk between six successive landmarks (e.g. cat, pool, chair) in an open environment; their virtual avatar’s eye height was approximately 180 cm above the ground, taking an egocentric view upon the virtual world. They were instructed to practice using the beacon during these six trials.

After completing the practice, participants were placed into the urban VE. The environment was unfamiliar to the participants and they received no information about it before the wayfinding task. Participants were instructed to use all available information to find each destination and were not specifically told to move quickly or efficiently through the environment. Movement controls were the same as in the practice task. They began the task directly facing their first landmark goal (Laundromat) and were instructed to “*Find the Laundromat*.” Once they navigated to within a 1-m radius of a labelled sign positioned in front of the landmark, the sequence of 40 trials began. Trial order was fixed across participants and each landmark was used twice as a destination (except for one landmark, the market); trials were of variable length and complexity. The overall route visited the sequence of 40 landmarks in a fixed pseudo-random order with a minimum of five landmark destinations occurring before being instructed to revisit a landmark. During each of the 40 trials, the participants could request up to five floating beacons to help orient them toward the current destination’s general direction. They requested a beacon by pressing the spacebar and it remained on-screen for 5 s; participants could continue to move during this time. The beacon always pointed in the direction of the current destination and if it was outside of the participant’s current field of view a red arrow pointed left or right to indicate the beacon’s location. After the 5 s elapsed, the beacon disappeared; there was no minimum request interval, so beacons could be requested immediately after one another. As depicted in Fig. 1b, the software always noted the number of beacons remaining for use and the current destination goal; it also depicted a compass rose in the upper right corner. Upon arrival at each successive destination, the next trial was triggered (e.g. “*You have found the Laundromat. Now find the Pet Store.*”). Once the participant successfully navigated between all 40 target destinations, the task finished. Finally, they completed the individual differences tasks and questionnaires.

### Data scoring

For the card rotation and road map tasks, we assessed accuracy. For the Santa Barbara Sense of Direction questionnaire, we used standard procedures to calculate sense of direction score for each participant (Hegarty et al., 2002). Note that scoring was reversed such that higher scores indicate higher sense of direction. For the Measure of Environmental Spatial Strategies, we used existing scoring methods to compute reliance on egocentric and survey strategies, and the use of cardinal points.

Recall that the navigation software automatically outputted location, orientation/yaw, and whether the participant was within or outside of an intersection; an intersection was defined as the polygon connecting the corners of surrounding buildings. Specifically, a participant entered an intersection when they moved within vector thresholds connecting the corners of buildings on each side of the intersection. All intersections were similarly-sized, though some were T-intersections (three-way) and some were four-way intersections. It also used markers to indicate the onset of a beacon request. Our primary questions regarding beacon request timing, whether requested within or outside of intersections, and preceding versus departing an intersection, were answered using these data alone. We also assessed wayfinding effectiveness by calculating path efficiency. As in our recent work, we chose to use path efficiency due to its relative invulnerability to stopping behavior, and the fact that path and time efficiency are typically highly correlated (Brunyé et al., 2014, 2016a; Brunyé, Gardony, Mahoney, & Taylor, 2012; Brunyé, Wood, Houck, & Taylor, 2016b). The present dataset was no exception, with highly correlated time and path efficiency measures (all Pearson *r* ≥ 0.90). To calculate path efficiency, we compared each origin-to-destination path to the optimal path determined via the A* algorithm (Hart, Nilsson, & Raphael, 1968). The optimal path length characterized the shortest route from an origin to destination. Path efficiency divided the optimal path length by the actual path length produced by the participant. In this manner, the highest attainable path efficiency was 1, with a minimum infinitely approaching 0. Note that across both experiments, effect sizes are provided using Cohen’s d or eta-squared.

## Experiment 1 results

### Overall beacon use

Overall, participants used at least one beacon on 96.8% of all trials (1936/2000) and they used about half of their allotted beacons overall (*M* = 2.29, *SD* = 1.05). Beacon use varied as a function of trial number, as verified in a within-participants analysis of variance (ANOVA), *F*(39, 1911) = 13.45, *p* < 0.001, η^2^ = 0.22. In general, certain trials elicited fewer beacon requests than others (range *M* = 1.26–3.0), but there was no significant correlation between mean beacon request frequency and trial number, *r*(40) = 0.26, *p* = 0.11. In other words, some trials seemed to elicit more beacon requests than others, but overall beacon requests did not increase or decrease consistently as trials progressed. We also tested whether beacon use on individual trials was predicted by path complexity in terms of path length, number of turns, and number of intersections. A linear regression model demonstrated that path length (β = 0.6, *p* < 0.001) and number of turns (β = 0.34, *p* = 0.028) significantly and positively predicted the number of beacons used, *F*(3, 39) = 15.8, *p* < 0.001, *R*^2^ = 0.57 (number of intersections did not predict beacon use, *p* = 0.83). Note that there was no significant correlation between mean frequency of beacon use and path efficiency (which was overall medium to high; mean path efficiency = 0.79), *r*(50) = 0.06, *p* = 0.69.

### Beacon use and intersections

Overall, when participants requested a beacon, they tended to do it more frequently in path segments (82% of requests; 3758/4591) than in intersections (18% of requests; 833/4591). The frequency of path segment requests exceeded that of chance, confirmed in a chi-square comparing frequency of beacons requested within versus outside of intersections, relative to area-weighted expected frequencies, χ^2^(1) = 442.9, *p* < 0.0001. Note that area-weighting considered the proportion of navigable (i.e. non-building) area occupied by intersections (33%) versus path segments (67%).

The majority (92.5%; 3475/3758) of beacon requests occurring outside of intersections occurred before entering an intersection (within the next 10 s). This pattern exceeded that of chance, confirmed in a chi-square comparing frequency of beacon requests followed by (versus not followed by) an entrance into an intersection within a 10 s period, χ^2^(1) = 6332.4, *p* < 0.0001. Again, this test used area-weighted expected values.

**Fig. 2:**
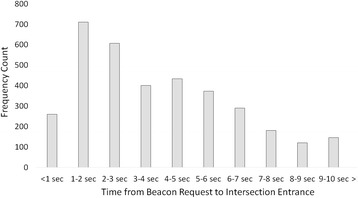
*Histogram* depicting the frequency of beacon requests resulting in an intersection entrance within the next 1 to 10 s

Figure 2 depicts a histogram of the overall pattern of beacon requests relative to intersections. In Fig. 2, we plot the frequency of beacon requests that resulted in entering an intersection within the next 1 to 10 s. The pattern shows that, on average, participants tended to request a beacon after leaving an intersection and before entering an intersection over the next 10 s of navigation. Approximately half of the beacon uses were associated with an entrance into an intersection within the first 4 s and the other half within the remaining 6 s; we explore possible predictors of this variability in the next section.

If wayfinders are using the beacon to enable decision-making before entering an intersection, then they should be faster to execute the behavior (go straight or turn) once they move into the intersection. To examine this possibility, we measured the amount of time wayfinders spent in an intersection after having activated versus not having activated a beacon during the 10 s before entering the intersection. For this analysis, we ensured that the beacon was off upon entering the intersection and was not requested while in the intersection; this resulted in the analysis of approximately 67% of all intersection entrances. We found significantly shorter time (in milliseconds) spent in an intersection when preceded by a beacon request (*M* = 935.4, *SD* = 161.9) than when not preceded by a beacon request (*M* = 1229.2, *SD* = 423.4), *t*(48) = 5.66, *p* < 0.001, Cohen’s d = 0.81. Overall, 80% of all participants (40/50) showed this numerical pattern.

### Individual differences: exploratory analyses

Table 1 includes a correlation matrix relating our individual differences measures and navigation behavior measures. Individual differences measures included accuracy and response times on the card rotation and road map tasks, composite spatial sense of direction scores, and spatial strategy scores (egocentric, survey, cardinal). A few primary results are worth mentioning. First, higher spatial sense of direction (SBSOD), road map accuracy, and spatial strategy scores were related to higher path efficiency. Second, performance on the road map task was positively associated with longer times between beacon request and entering an intersection. Of course, while our individual differences measures were mostly restricted to spatial measures, we do not intend to suggest that our results are not also driven by differences in non-spatial abilities and strategies.

**Table 1:** Experiment 1 correlation matrix relating individual differences measures and behavior outcomes

	Card rotation accuracy	Card rotation RTs	FRS egocentric	FRS survey	FRS cardinal	Road map accuracy	Road map RTs	SBSOD	Beacon frequency	Beacon use outside intersect. (%)	Beacon use outside then enter (%)	Time until entering intersection	Path efficiency
Card rotation accuracy	1												
Card rotation RTs	0.316^a^	1											
FRS egocentric	0.119	0.150	1										
FRS survey	0.179	0.093	0.684^b^	1									
FRS cardinal	−0.085	−0.138	0.452^b^	0.410^b^	1								
Road map accuracy	0.391^b^	0.066	0.049	0.215	−0.061	1							
Road map RTs	0.130	0.456^b^	−0.139	−0.131	−0.038	0.186	1						
SBSOD	−0.064	0.117	0.706^b^	0.601^b^	0.450^b^	0.210	−0.119	1					
Beacon frequency	0.006	−0.078	−0.238	0.079	−0.017	0.097	−0.089	−0.170	1				
Beacon use outside intersections (%)	−0.274	−0.023	0.153	0.099	0.177	0.054	0.049	0.271	0.115	1			
Beacon use outside, then inside < 10 s	0.027	−0.009	−0.193	−0.014	0.115	−0.172	0.012	−0.193	0.240	−0.218	1		
Time until entering intersection	−0.064	0.085	0.212	0.042	0.233	0.338^a^	0.318^a^	0.302^a^	−0.165	0.269	−0.440^b^	1	
Path efficiency	0.069	−0.045	0.411^b^	0.390^b^	0.341^a^	0.337^a^	−0.142	0.473^b^	0.058	0.083	−0.165	0.409^b^	1

^a^Correlation is significant at the 0.05 level (2-tailed)

^b^Correlation is significant at the 0.01 level (2-tailed)

We also used t-tests to examine whether our two categorical individual differences (gender, video gaming experience) were related to navigation behavior. Male participants tended to request beacons earlier on approach to an intersection (*M*_*male*_ = 3.49, *SD*_*male*_ = 0.36) than women (*M*_*female*_ = 3.19, *SD*_*female*_ = 0.37), *t*(48) = 2.58, *p* = 0.013, Cohen’s d = 0.86. Men also had marginally higher (*M*_*male*_ = 0.80, *SD*_*male*_ = 0.06) overall path efficiency than women (*M*_*female*_ = 0.76, *SD*_*female*_ = 0.05), *t*(48) = 2.15, *p* = 0.037. Finally, there was some suggestion that video gamers showed lower mean beacon use (*M* = 2.02, *SD* = 0.4) relative to non-video gamers (*M* = 2.37, *SD* = 0.64), *t*(35) = 2.06, *p* = 0.047.

## Experiment 1 discussion

Our first experiment demonstrated that when participants could request a beacon to inform a decision, they tended to make the request within path segments, not intersections. There was no task-related incentive for this behavior; participants could willingly pause their walking within an intersection and they were not instructed to move between destinations quickly. Furthermore, when the beacon was requested in a path segment, participants showed a tendency to subsequently enter an intersection within the next few seconds and to navigate through it more efficiently than when they did not request a beacon before entering. Finally, a longer duration between a beacon request and the entrance into an intersection was generally associated with higher spatial skills, suggesting those with higher spatial skills made decisions earlier than others. Overall, these data provide evidence that information-seeking behavior to inform a decision occurs, in many cases, before entering an intersection.

## Experiment 2 introduction

Our second experiment was designed to test whether evidence for spatial decisions occurring before entering an intersection would be reflected in wayfinders’ movement patterns. As wayfinders developed knowledge over the course of trials, we asked whether their movement dynamics on approach to intersections show evidence of advanced decision-making. Specifically, we asked whether wayfinders would begin to head in the direction of an upcoming turn. Wayfinders may begin to move toward their upcoming turn direction, placing themselves in a more efficient position before entering an intersection. We expect this type of behavior to emerge as wayfinders gain increased familiarity with the environment and are better able to plan upcoming turns. If this pattern holds true, it would provide strong evidence that spatial decision-making is a dynamic process occurring far in advance of an intersection, particularly as familiarity with the environment increases. To avoid any influence of beacons on heading direction changes, this experiment asked participants to navigate and learn the environment without navigation guidance. Like in Experiment 1, we conducted some exploratory correlations to examine whether individual differences would predict any effects.

## Experiment 2 method

### Participants and design

Forty-one individuals participated for monetary compensation; nine participants failed to complete the task due to mild simulator sickness (n = 3) or difficulty performing the wayfinding task (i.e. not completing the task within 1.5 h; n = 6). The resulting sample consisted of 17 men and 15 women (*M*_age_ = 21.5). Each participant completed a sequence of 20 wayfinding trials, in a within-participants design. A pilot study determined that navigating 20 trials was attainable in a roughly 1-h session; using 40 trials (i.e. as in Experiment 1) showed an exceedingly high failure rate. All procedures were approved by the institutional review boards at Tufts University and the U.S. Army.

### Materials

#### Individual differences measures

We collected data on the same set of questionnaires and tasks used in Experiment 1: the card rotation task, road map task, Santa Barbara Sense of Direction, spatial strategy questionnaire, and the video game questionnaire.

#### Virtual environment

The same large-scale urban desktop virtual environment was used as in Experiment 1, but no beacon-based navigation aid was available.

### Procedure

Participants visited the laboratory for an approximately 1-h session. All characteristics of the practice session and navigation control schemes were identical to Experiment 1. All procedures for the primary navigation task were identical to those used in Experiment 1, except for completing 20 rather than 40 trials, and no beacon availability.

### Data scoring

We calculated path efficiency in the same manner as in Experiment 1. To assess whether wayfinders oriented themselves toward an upcoming turn direction, we evaluated heading direction as a function of samples (time) preceding an entrance into an intersection. To do so, we coded all instances of intersection entrances and exits by whether they occurred from the north, south, east, or west. Using circular statistics (Berens, 2009), we then calculated mean heading separately for instances involving a left turn, right turn, continuing straight, or turning around. This allowed us to consider heading direction during the 5-s of time preceding and 5-s of time following an intersection entrance, with reference to the resulting intersection-related behavior. Circular statistics transform angular data to vectors, allowing for the analysis of angular data that fall on a circular (or directional) distribution with no true zero and an arbitrary designation of low and high values (e.g. orientation data in the range of 1°–360°). In these cases, data cannot be analyzed with traditional statistics; for instance, averaging two very similar orientations (e.g. 2° and 358°) would not result in a rational result (e.g. 180°).

## Experiment 2 results

### Overall path efficiency

Path efficiency was low to moderate overall (*M* = 0.37, *SD* = 0.11), though it did increase significantly from the first set of ten (*M* = 0.33, *SD* = 0.1) to the second set of ten (*M* = 0.41, *SD* = 0.11) trials, *F*(1, 31) = 18.11, *p* < 0.001, η^2^ = 0.37. Participants also tended to spend less time in intersections during the second set of ten trials (*M* = 1696 ms, *SD* = 753) versus the first set of ten trials (*M* = 1950 ms, *SD* = 779), *F*(1, 31) = 23.51, *p* < 0.001, η^2^ = 0.43.

### Intersections and heading direction

**Fig. 3:**
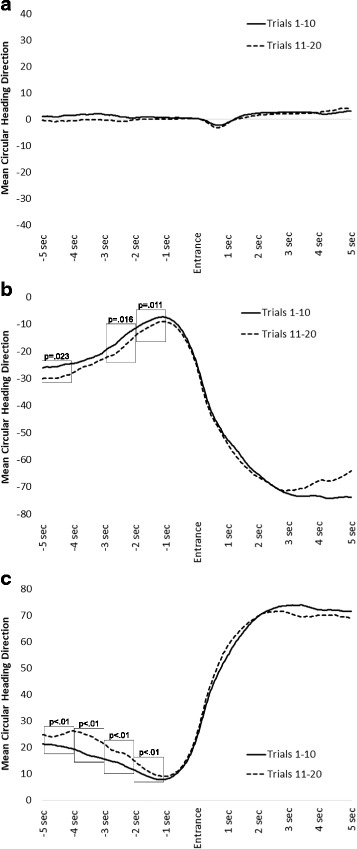
**a**–**c** Mean circular heading direction for the two Trial Sets (trials 1–10 vs 11–20) and 10 time bins (five preceding and five following an intersection entrance). 0 degrees indicates north heading. Data are depicted for all instances when a wayfinder continued straight (**a**), turned left (**b**), and turned right (**c**). Significant differences within examined time bins are indicated by *rectangles* with associated *p* values

Overall, there were 24,604 instances of entering and exiting an intersection, an average of about 769 for each participant. Continuing straight occurred most frequently (10,460; 43%), followed by turning left (7479; 30%), turning right (6423; 26%), and then turning around (242; < 1%). Given the low frequency of turn-around behaviors, these instances were not considered for analysis. Mean heading direction data are depicted in Fig. 3a–c, for continuing straight, and left and right turns, respectively.

For continuing straight (Fig. 3a), notice how mean heading direction is very similar for both sets of trials (1–10 versus 11–20). This is the case for the 5 s preceding and following an intersection entrance. To confirm no differences preceding entrance into the intersection, we used a repeated-measures analysis of variance (ANOVA) with two factors: Trial Set (two levels: 1–10, 11–20), and Time Bin (10 levels: 1-s increments preceding and following intersection entrance). This allowed us to compare mean headings for trials 1–10 versus 11–20 in each of the time bins. There was no significant interaction between Trial Set and Time Bin, *F*(9, 279) = 1.25, *p* = 0.27, η^2^ < 0.01, demonstrating that heading direction did not vary over time as a function of Trial Set.

For left turns (Fig. 3b), notice how executing a left turn at the intersection results in mean heading of approximately − 70°. This is because the participant turned left in the intersection: more negative values indicate relatively leftward headings. During later trials, wayfinders showed a tendency to orient themselves toward more leftward (negative values) angles in the direction of the upcoming turn. This was reflected in a significant interaction between Trial Set and Time Bin, *F*(9, 279) = 8.37, *p* < 0.001, η^2^ < 0.01. To test for differences between Trial Sets over time, we used simple effects ANOVAs to compare mean headings for trials 1–10 versus 11–20, in each of five 1-s time bins preceding the intersection. The difference reached significance in time bins 1 (*p* = 0.023), 3 (*p* = 0.016), and 4 (*p* = 0.011). It did not reach significance in time bin 2 (*p* = 0.14) or in time bin 5 (*p* = 0.08). Thus, there was some evidence for orienting toward the direction of an upcoming left turn, particularly during later trials. If this pattern is indicative of knowledge development, it should also correlate with path efficiency gains during later trials. To examine this question, we calculated mean orientation difference scores comparing trials 11–20 versus 1–10 (for time bins 1–5) and mean path efficiency scores comparing trials 11–20 versus 1–10. In this manner, more negative mean orientation difference scores indicate a relative tendency to orient toward an upcoming left turn during later trials, and more positive path efficiency scores indicate higher path efficiency during later trials. Thus, a negative correlation would suggest that a relative tendency to orient toward an upcoming turn direction on later trials is related to relatively high path efficiency on those trials. This pattern was found, *r*(32) = −0.42, *p* = 0.017.

For right turns (Fig. 3c), notice how executing a right turn at the intersection results in mean heading of approximately 70°. This is because the participant turned right in the intersection: more positive values indicate relatively rightward headings. During later trials, wayfinders showed a tendency to orient themselves toward more rightward (positive values) angles in the direction of the upcoming turn. This was reflected in a significant interaction between Trial Set and Time Bin, *F*(9, 279) = 10.65, *p* < 0.001, η^2^ < 0.01. To test for differences between Trial Sets over time, we used simple effects ANOVAs to compare mean headings for trials 1–10 versus 11–20, in each of five 1-s time bins preceding the intersection. The difference reached significance in time bins 1–4 (*p* values < 0.01), but not in time bin 5 (*p* = 0.17). As with left turns, we asked whether this pattern is correlated with path efficiency gains during later trials. For right turns, a positive correlation would suggest that a relative tendency to orient toward an upcoming right turn on later trials is related to relative high path efficiency on those trials. This pattern was found, *r*(32) = 0.35, *p* = 0.048.

### Individual differences: exploratory analyses

We conducted a series of exploratory analyses to assess the possible relationships between individual differences and the extent to which wayfinders showed headings directed toward an upcoming turn direction. To do so, we calculated difference scores for the two turn directions (left, right) by subtracting mean values from trials 1–10 from mean values from trials 11–20, across the five time bins. The absolute values of these two difference scores were then averaged to create a single value reflecting the magnitude of heading change as a function of experience in the environment. Higher values thus reflected a tendency during later trials to adjust heading in the direction of an upcoming turn, collapsed across left and right turn directions.

Table 2 details a correlation matrix relating individual differences to the heading change difference score and path efficiency. Higher spatial sense of direction and spatial strategies were associated with higher orientation difference scores and path efficiency. In other words, those with higher spatial sense of direction, and those who show higher spatial strategies, tended to show a stronger tendency to head in the direction of an upcoming turn during later trials, and higher path efficiency.

**Table 2:** Experiment 2 correlation matrix relating individual differences measures and behavior outcomes

	Card rotation accuracy	Card rotation RTs	FRS egocentric	FRS survey	FRS cardinal	Road map accuracy	Road map RTs	SBSOD	Orientation change difference	Path efficiency
Card rotation accuracy	1									
Card rotation RTs	0.332	1								
FRS egocentric	0.090	0.141	1							
FRS survey	−0.004	−0.074	0.695^b^	1						
FRS cardinal	−0.025	−0.078	0.358^a^	0.432^a^	1					
Road map accuracy	0.734^b^	0.447^a^	0.016	−0.014	−0.182	1				
Road map RTs	0.477^b^	0.326	0.126	0.064	0.046	0.605^b^	1			
SBSOD	0.095	0.094	0.803^b^	0.653^b^	0.470^b^	0.052	0.305	1		
Orientation change difference	−0.250	−0.178	0.489^b^	0.437^a^	0.425^a^	−0.322	−0.149	0.543^b^	1	
Path efficiency	0.251	−0.165	0.451^b^	0.402^a^	0.562^b^	−0.019	0.079	0.480^b^	0.383^a^	1

^a^Correlation is significant at the 0.05 level (2-tailed)

^b^Correlation is significant at the 0.01 level (2-tailed)

Unlike in Experiment 1, gamers and non-gamers, and men and women, showed similar path efficiency and a similar tendency to head in the direction of an upcoming turn during later trials (all *p* values > 0.23).

## Experiment 2 discussion

Our second experiment demonstrated that as wayfinders develop knowledge over the course of environmental experience, their movement dynamics are suggestive of decision-making before entering an intersection. Specifically, wayfinders showed a tendency to orient their bodies toward a resulting turn direction several seconds before arriving at the intersection. Assuming translational movement during these times, it is likely the case that these orientation changes corresponded to wayfinders maneuvering their avatars toward the side of the road corresponding to their chosen turn direction, to maximize turn efficiency once entering the intersection. Complementing Experiment 1, we provide strong evidence that decisions on whether to turn or continue straight are made far in advance of an intersection, particularly when navigating a familiar environment. In Experiment 1, this was demonstrated by examining information-seeking behavior before entering an intersection and in the present experiment it was demonstrated in dynamic movement behavior before entering an intersection. We also found evidence that these patterns are partially predicted by spatial skills and strategies; higher spatial skills and reliance on particular strategies were associated with more evidence of making turn decisions prior to entering an intersection.

## General discussion

Empirical research and theory are equivocal regarding whether wayfinders make decisions within or before entering an intersection and how this process might be affected by environmental experience. The present study found support for a dynamic, protracted spatial decision-making process that occurs in anticipation of entering an intersection. In Experiment 1, when participants could request a beacon to inform a decision, they tended to make the request within path segments, not intersections. Furthermore, when the beacon was requested in a path segment, participants showed a tendency to subsequently enter an intersection within the next few seconds. In Experiment 2, participants tended to show dynamic heading changes predictive of a subsequent turn direction, suggesting they were preparing for a turn response before arriving in an intersection. In both experiments, we also found evidence that information-seeking and movement behaviors preceding an intersection were generally associated with higher spatial skills, suggesting those with higher spatial skills made decisions earlier than others.

Beacon request data from Experiment 1 demonstrated that most requests were made while participants were navigating between intersections, on path segments. Presumably, participants were attempting to gather environmental information, in this case the general direction of a destination, to identify whether a turn was required, and deciding at which intersection it might be most efficient. There are several possible cognitive mechanisms that could be updated by the presence of a beacon. First, wayfinders may activate a beacon to disambiguate whether to continue straight or turn in a particular direction, in order to move closer to the ultimate destination (Cornell, Heth, & Alberts, 1994). Second, when several forthcoming turn options exist, wayfinders may activate a beacon in order to disambiguate which intersection to use (Christenfeld, 1995). Third, wayfinders may request a beacon to alleviate stress that accumulates as a result of decision uncertainty during navigating (Hund & Minarik, 2009; Lawton & Kallai, 2002). Finally, wayfinders may activate a beacon to help orient themselves relative to the goal direction; this process may be particularly useful following a series of turns when dead reckoning (path integration) becomes more cognitively demanding. In these cases, the beacon may serve to reduce this cognitive burden by quickly updating an egocentric representation (Burgess, 2006; Kelly & McNamara, 2008; Wang, 1999; Wang & Spelke, 2000). Thus, using a beacon to gather information during wayfinding is likely in the service of decision efficiency, whether identifying turn options and directions, or updating mental representations.

Further evidence that beacons provided information to afford decision-making and a subsequent intersection-related behavior comes from the time-course of beacon use on approach to an intersection. Most beacon requests occurring outside of intersections were followed by an arrival in an intersection within the next 10 s. While most trials showed an entrance into the intersection within 4 s of beacon onset, approximately half of trials showed an entrance up to 10 s after beacon onset. Recall that upon request, the beacon remained on-screen for 5 s. Thus, the beacon could be used to simply guide locomotion through an intersection if it were still on-screen during the intersection entrance; this process can occur without any internal representation of the environment, simply moving toward the visible beacon (Montello, 2001). Cases where the beacon was off-screen by the time the intersection was entered present more compelling evidence that the decision on whether to continue straight or turn was likely already made before the intersection was entered. This possibility is supported by our analysis of behavior while in intersections. Specifically, participants spent significantly less time, on average, in an intersection when they saw a beacon within the past 10 s of navigation. Critically, in these cases the beacon was no longer on-screen by the time the wayfinder was in the intersection. Thus, having seen a beacon before entering an intersection allowed the wayfinder to formulate a plan, and then efficiently execute the plan once in the intersection. Thus, in many cases it seems that upcoming intersections prompt information-seeking behavior and decision-making, such that once the intersection is reached a planned behavior can be efficiently executed.

Experiment 2 complemented these findings by demonstrating that movement behaviors suggest a dynamic decision-making process in advance of entering an intersection. Practically speaking, pedestrian wayfinders moved themselves to the side of the road (and onto the sidewalk) toward their ultimate turn direction, likely to maximize the efficiency of in-intersection maneuvers. This pattern is analogous to the way that robots can evolve to use cognitive strategies and efficiently move toward the side of a hallway corresponding to a subsequent turn direction (Carvalho & Nolfi, 2016). It is also related to the timing of GPS prompts, wherein users find utility in planning a behavior long before its execution is required. GPS devices use navigation prompts (e.g. *In 500 ft, turn right onto Main St.*) that are intentionally delivered before entering an intersection, typically using an algorithm that provides a maneuver instruction at a variable warning distance from a junction (Tamai & Pai, 1999). In other words, the systems intentionally provide information to enable planning and decision-making regarding an upcoming maneuver (Brown & Laurier, 2012; Rehrl, Häusler, Leitinger, & Bell, 2014). When these devices fail to provide information in a timely manner, the planning and decision-making process is interrupted, possibly resulting in a failed maneuver. Advanced planning, whether relying upon emergent environmental memory or a GPS device, provides the wayfinder with the opportunity to maneuver themselves to be in an optimal position for an approaching turn. The notion that wayfinders make decisions in advance of an intersection approach also supports some research from driving, demonstrating that drivers often decide whether to continue or stop far in advance of arrival at a signalized intersection (Gates, Noyce, Laracuente, & Nordheim, 2007; Yang, Tian, Wang, Zhou, & Liang, 2014).

Across experiments, we also explored how individual differences in spatial abilities and strategies might be associated with wayfinding outcomes. In Experiment 1, we found some evidence that higher accuracy on the road map test was associated with using a beacon farther from an approaching intersection. The road map test assesses the ability to switch perspectives from an allocentric map view to making egocentric orientation judgments (Hegarty et al., 2002; Schultz, 1991). In the “Introduction” section, we referred to this task as demanding the perspective transformation skill. It could be the case that those with higher perspective transformation skills are more capable of making and maintaining egocentric judgments before making a spatial decision. Experiment 2 results complemented these findings, demonstrating a positive association between spatial skills and advance decision-making. Across experiments we also found evidence that higher overall path efficiency was associated with several individual differences measures, such as higher sense of direction, greater road map accuracy, and varied strategies for thinking about and navigating environments (i.e. egocentric, allocentric). None of these results was particularly surprising, though they do converge with recent evidence suggesting the importance of individual differences in spatial strategy for understanding wayfinding performance (Münzer, Fehringer, & Kühl, 2016).

### Informed versus uninformed search

Some wayfinding research proposes that searching for a destination can be usefully divided into informed versus uninformed searches (Ruddle et al., 1999; Wiener et al., 2009). In an informed search, the wayfinder has in-depth environmental knowledge, such as a cognitive map or other mental representation of environmental layout. The wayfinder uses this stored knowledge to plan and execute routes, recognize landmarks, and orient themselves in the environment. Thus, searching for a novel destination is continually informed and guided by this environmental knowledge. A large body of wayfinding research and theory tends to focus on informed search (Gärling & Gärling, 1988; Ruddle et al., 1999; Wiener et al., 2004, 2009), though uninformed search is characteristic of many real-world wayfinding behaviors (e.g. tourism, search and rescue, disaster-relief efforts, military operations).

In an uninformed search, the environment is not familiar to the wayfinder, and thus the entirety of the problem space is unknown. As noted by Wiener et al., uninformed search is inherently more complex and mentally taxing for a wayfinder (Wiener et al., 2009), because the wayfinder can only coarsely plan a search for a destination (e.g. go north), and attention must be allocated to monitoring the environment, path integration, and updating any search strategy. The present task is best categorized as an uninformed search for destinations, given the lack of environmental knowledge at the onset of the wayfinding task. Of course, as experience with the environment unfolds, an emergent representation is formed and relied upon, as seen in the Experiment 2 results. We suggest that decisions may be more likely to occur while a wayfinder is within an intersection during an uninformed search, as they perceive the environment and attempt to find visual cues that will help them orient themselves. This process may be best served by pausing in an intersection to look in multiple directions and possibly recognize salient distal landmarks (Denis, Mores, Gras, Gyselinck, & Daniel, 2014; Klippel et al., 2003; Klippel & Winter, 2005). As experience with the environment grows, we suggest that the task becomes more like an informed search (i.e. knowing goal locations, paths toward goals, and environmental layout) and wayfinders show evidence of planning turns before entering an intersection. This suggestion converges with research examining wayfinding to a known target, which implies that planning the entirety of a route can occur before beginning a journey (Allen, 1999). In some cases, the route may be precisely planned, including a sequence of turns toward a destination, whereas in other cases certain strategies may emerge that dictate particular turns. For instance, a lawnmower or perimeter search strategy will dictate turn directions at particular intersections (Buechner, Holscher, & Wiener, 2009).

### Applied implications

We motivated this research by discussing innovative navigation guidance systems that might hold potential for reducing some of the resource requirements and negative consequences of conventional turn-by-turn navigation systems. We focused here on beacon-based systems, which can provide visual or vibrotactile guidance by indicating the general direction and distance to a destination. These systems are envisioned to reduce some of the attentional burden of turn-by-turn guidance, help wayfinders maintain active attention toward and engagement with their environments, and reduce some of the negative memory consequences of conventional navigation systems. Whereas the promise of such systems is great, there is a lack of information regarding when and where in an environment these systems should be used for pedestrian navigation. This is especially relevant given the recent emphasis on developing adaptive systems that modulate their cueing based on the circumstances surrounding system use (Doswell & Skinner, 2014; Fuchs & Schwarz, 2017; Scerbo, Freeman, & Mikulka, 2003). Thus, in addition to asking relatively basic research questions regarding the dynamics of spatial decision-making, the present research also provides initial data outcomes relevant for informing the development of beacon-based navigation systems.

The present results can be applied to the development of beacon-based navigation systems in three primary ways. First, we demonstrate that visual beacons can be used to guide efficient navigation through dense urban virtual environments, suggesting future potential for these systems (Brunyé et al., 2016a; Loomis, Golledge, & Klatzky, 1998). Second, pedestrians seem to rely upon these systems during the approach to an intersection, suggesting that adaptive future systems would find value in portraying beacons before pedestrians arrive in an intersection, affording them the opportunity to plan maneuvers in advance. This suggestion is supported by Experiment 2 results, which further demonstrate that wayfinders move in ways that demonstrate anticipatory planning and behavior before entering an intersection. It is also supported by anticipatory *driver* behavior during approach to signalized intersections (Yang et al., 2014). Third, we demonstrate that the dynamics of wayfinding behavior and beacon system reliance are contingent on not only location relative to an intersection, but also interactions between the user’s characteristics (e.g. spatial skills) and environmental experience. This finding complicates the relationship between humans and navigation systems, suggesting that systems adaptive to not only location but also users and their relationship with environments might prove beneficial to guiding navigation (Fuchs & Schwarz, 2017). Our research thus reveals a layer of complexity motivating continuing research regarding human-systems interaction during wayfinding.

### Limitations

In Experiment 1, due to the novel nature of the environment, the wayfinder is likely more reliant upon guidance to remain oriented toward the goal destination and avoid getting lost (Wiener et al., 2009). Though our second experiment provided an opportunity to learn the environment through experience, and we find ample evidence that spatial decisions likely unfold prior to entering an intersection, we cannot speak to the decision-making process in a highly familiar environment (e.g. a home city). We might expect that with fully informed search, much of the decision-making is made during an initial planning phase rather than iteratively during the wayfinding process. Of course, a plan could be perturbed by changes in destination, deadlines, detours, or other path obstructions, but these circumstances remain to be explored. We also cannot speak to whether beacon requests prior to entering an intersection directly affect within-intersection behavior, as beacon availability was never directly manipulated. In our continuing and relatively applied research, we are automatically triggering beacon delivery in response to approaches to an intersection. By triggering the beacon at a variable distance from the intersection, we can directly examine the effect of beacon timing on within-intersection behavior.

The present research used desktop VEs to explore the time-course of spatial decision-making, though there is some debate whether VEs are a useful surrogate for research in real-world environments. Several studies have demonstrated that people show high transfer of environmental knowledge from desktop or immersive virtual environments to real worlds, such as transferring route knowledge (Bliss & Tidwell, 1995; Witmer, Bailey, Knerr, & Parsons, 1996) or knowledge of directions, distances, and layouts (Richardson, Montello, & Hegarty, 1999; Ruddle et al., 1999). For instance, Waller et al. demonstrated that performance navigating a real-world environment was similar following virtual or real-world environment experience, and virtual environment experience was better than experience studying a map only (Waller, Hunt, & Knapp, 1998). However, desktop VEs also limit kinesthetic and vestibular sensing (and whole-body movement) that is very important for real-world spatial learning (Taube, Valerio, & Yoder, 2013). For instance, physical body rotation is important for egocentric updating (Klatzky, Loomis, Beall, Chance, & Golledge, 1998; Riecke et al., 2010; Ruddle & Lessels, 2006) and proprioceptive information available while walking a route is important for route learning (Gale, Golledge, Pellegrino, & Doherty, 1990). Thus, while present research provides some evidence that spatial decision-making may occur before entering an intersection, it is unclear how whole-body movement during real-world navigation might interact with our results (Gardony et al., 2013, 2015). Our continuing research explores these questions while pedestrians navigate unfamiliar real-world neighborhoods with guidance presented via heads-up displays or vibrotactile belts.

### Conclusions

In a recent article, Montello proposes that certain elements of environments, such as landmarks, are exaggerated in their value for guiding wayfinding decisions and behavior (Montello, 2017). A central tenet of this proposal is that when empirical research and theory focus on the importance of a specific environmental element, it overly simplifies and undervalues other important processes such as decision-making, reasoning, and developing and updating spatial representations. The present research suggests that wayfinding research sometimes oversimplifies and undervalues the importance of a dynamic decision-making process that unfolds during wayfinding. While intersections may prompt a decision, and elicit overt behavior that reflects a decision, the process of arriving at a spatial decision often occurs before arriving in the intersection. This finding reveals complex spatial decision dynamics during pedestrian wayfinding and also provides a data foundation for developing future adaptive navigation guidance systems.

## Abbreviations

ANOVA: Analysis of variance
GPS: Geographical positioning system
VE: Virtual environment
